# Properties of Geopolymers Based on Fly Ash with the Addition of Asphalt from Road Surface Demolition

**DOI:** 10.3390/ma18194488

**Published:** 2025-09-26

**Authors:** Barbara Kozub

**Affiliations:** 1 Department of Materials Engineering, Faculty of Materials Engineering and Physics, Cracow University of Technology, Jana Pawła II 37, 31-864 Cracow, Poland; barbara.kozub@pk.edu.pl; 2 Interdisciplinary Center for Circular Economy, Cracow University of Technology, Warszawska 24, 31-155 Krakow, Poland

**Keywords:** geopolymer, fly ash, recycled asphalt, quartz sand, construction composites

## Abstract

This article presents the results of a comprehensive investigation into geopolymer composites synthesized from fly ash, incorporating ground asphalt derived from reclaimed road pavement and quartz sand. The primary objective of this study was to elucidate the influence of mixture composition on the mechanical, physical, and microstructural characteristics of the developed materials. The innovative aspect of this research lies in the integration of two distinct filler types—mineral (quartz sand) and organic-mineral (milled asphalt)—within a single geopolymer matrix, while preserving key performance parameters required for engineering applications, including compressive and flexural strength, density, water absorption, and abrasion resistance. The experimental methodology encompassed the characterization of the raw materials by X-ray diffraction (XRD), chemical composition analysis via X-ray fluorescence (XRF), and assessment of particle size distribution. Additionally, the produced geopolymer materials underwent density determination, compressive and flexural strength measurements, abrasion testing, and mass water absorption evaluation. The chemical composition was further examined using XRF, and the surface morphology of the specimens was analyzed by scanning electron microscopy (SEM). The findings demonstrate that the incorporation of quartz sand enhances the density and mechanical strength of the composites, whereas the addition of recycled asphalt, despite causing a modest reduction in mechanical performance at elevated dosages, augments water resistance. Moreover, ternary composite material provide an optimal compromise between mechanical strength and durability under humid conditions. Overall, the results substantiate the feasibility of utilizing asphalt waste for the fabrication of functional and sustainable geopolymer materials suitable for construction applications.

## 1. Introduction

Geopolymers, categorized as inorganic polymers, are materials possessing an aluminosilicate framework that have garnered considerable attention in materials science research over the past three decades [1,2]. These materials are synthesized via the alkaline activation of raw materials rich in silicon and aluminum (aluminosilicate precursors), such as fly ash, metallurgical slag, or metakaolin. The synthesis process typically involves dissolving aluminosilicate sources in an alkaline solution, resulting in the formation of a three-dimensional network of silicate and aluminate polymer chains [3,4]. The process of manufacturing geopolymer materials not only promotes a low-carbon economy and sustainable development through the recycling of waste materials but also significantly lowers CO_2_ emissions compared to the production of traditional Portland cement [5,6]. Geopolymers exhibit several beneficial properties, including high mechanical strength, high-temperature resistance, low permeability, and good chemical resistance [1,2]. The properties of geopolymers are significantly influenced by several factors, including the type of raw materials used, curing conditions, and the presence of various additives. Numerous studies have shown that the introduction of additives in the form of complementary materials such as blast furnace slag [7], multiple types of fibers [8], including polypropylene [9,10], polyvinyl alcohol (PVA) [11,12,13], natural fibers such as hemp and flax [14,15,16], or steel fibers [17], increases the compressive strength and durability of geopolymer composites.

Furthermore, modification of curing temperature and duration can optimize the polymerization process, thereby enhancing the mechanical performance of the end product [18,19]. The selection of the alkaline activator is also pivotal in dictating the kinetics of geopolymerization and, by extension, the mechanical and physical attributes of the resultant geopolymer [20]. Among the most widely utilized activators is a combination of sodium hydroxide (NaOH) and sodium silicate (Na_2_SiO_3_). This activator facilitates the dissolution of the aluminosilicate phase in the precursors, supporting the polymerization process necessary to form the geopolymer network [21,22]. However, it should be remembered that the effectiveness of the activator used depends on its concentration and proportions [23,24,25]. Recent literature demonstrates the potential of using alternative materials, such as wood ash liquor and brewery sludge, as activators, thereby expanding the range of materials that can potentially support final geopolymer production [6,26]. These alternatives, in addition to reducing the costs associated with commercially available alkaline activators, contribute to the recycling of waste materials and make geopolymer technology more sustainable [27]. Fly ash-based geopolymers represent a significant advance in sustainable building materials, offering numerous advantages over traditional Portland cement. Fly ash, a byproduct of coal combustion in power plants, contains high concentrations of silica (SiO_2_) and alumina (Al_2_O_3_) and is one of the most commonly used geopolymer precursors [28,29,30]. The properties of fly ash-based geopolymers can vary significantly depending on the type of fly ash used. Depending on the calcium content, fly ash is divided into Class F (low-calcium) and Class C (high-calcium). Class F fly ash is predominantly amorphous material, which results in better reactivity and higher compressive strength compared to class C fly ash, which contains a large amount of calcium that can negatively affect the polymerization process [31,32].

Interest in combining geopolymers with other materials stems from both the desire to improve specific parameters and the need to manage industrial waste. In recent years, the incorporation of mineral and mineral-organic fractions from recycling into geopolymer matrices has become a significant trend [33,34,35,36]. Milled asphalt, consisting of mineral aggregates bound by bituminous material, is generated during pavement rehabilitation and represents a substantial waste stream. Although milled asphalt exhibits limited chemical reactivity, its incorporation into geopolymer matrices can markedly influence the physical properties, microstructure, and durability of the resulting composites [37]. A recent investigation by Albidah [38] evaluated the effects of substituting natural aggregate with recycled asphalt aggregate in metakaolin-based geopolymer concrete, both under ambient conditions and following exposure to elevated temperatures. A range of mixtures with varying proportions of natural aggregate replacement was examined. The study analyzed the effects on compressive strength, elastic modulus, strain at ultimate strength, as well as flexural strength and behavior after heating to 300 °C and 600 °C. The obtained predictive models corresponded well to the experimental results, confirming their usefulness in assessing the mechanical properties of such materials.

Combining fly ash with milled asphalt and sand in a single geopolymer matrix is an innovative approach. There are few studies in the literature that simultaneously analyze the effect of both additives on the mechanical and physical parameters of composites. Potential benefits include reduced raw material costs, improved performance, and the utilization of waste that would otherwise be landfilled.

This work aimed to design and investigate the properties of geopolymers produced from fly ash, supplemented with sand and asphalt from road demolition. The author aimed to examine the impact of introducing these components—one of which is a traditional mineral material and the other recycled waste—on the selected physico-mechanical and performance properties of geopolymer mixtures. A reference sample prepared solely from fly ash served as a benchmark for the research conducted. This work contributes to the search for sustainable material technologies. It aims to assess the potential for practical application of the tested composites in construction, following the principles of a circular economy.

## 2. Materials and Methods

### 2.1. Materials

Class F fly ash obtained from the Skawina CHP Plant (Poland) was utilized for the preparation of test specimens. Quartz sand and milled asphalt, recovered from the demolition of road surfaces, served as admixture materials. Each additive was incorporated at levels of 25% or 50% by weight relative to the fly ash.

To broaden the scope of the investigation and assess the interaction and evaluate the interactions between the additives, additional samples were prepared with ternary composite materials: (i) 50% fly ash, 25% sand, and 25% asphalt, and (ii) approximately 33% by weight of each of the three components. The proportions of quartz sand and milled asphalt were selected based on previous studies on geopolymer composites [39] and practical feasibility in pavement applications. The selected substitution range (25–50%) enabled the assessment of both moderate and high replacement scenarios.

The milled asphalt, as received, was not suitable for direct use in the geopolymer matrix. Before use, it was pretreated by crushing in a jaw crusher and then sieving to obtain a homogeneous fraction with the required grain size. The designations of all prepared mixture variants, along with the weight share of individual ingredients, are summarized in Table 1.

The liquid-to-solid (L/S) ratio in geopolymers is a critical parameter that significantly affects the material’s workability, density, and mechanical performance. Although there is no universal ‘optimum’ value, typical ranges for fly ash-based geopolymers are reported between 0.35 and 0.60 [1,2]. In this study, the L/S ratio was adjusted depending on the type and amount of additive. For the reference mixture (100% FA) and sand-modified systems, an L/S ratio of 0.40 was used (corresponding to 400 g of activator solution per 1000 g of dry ingredients). For asphalt-modified mixtures, a slight increase in solution volume was required due to the dryness and hydrophobicity of asphalt particles. Therefore, for 25% asphalt replacement, 425 g of activator solution was used per 1000 g of solids (L/S = 0.425), while for 50% asphalt replacement, 450 g of solution was applied (L/S = 0.45). For ternary composite materials containing both sand and asphalt, the liquid content was adjusted according to the asphalt proportion, i.e., equivalent to the mix with 25% asphalt (L/S = 0.425).

### 2.2. Sample Preparation

Geopolymer mixtures were prepared in a planetary mixer. The procedure commenced with the thorough blending of fly ash and the designated additives (sand and/or milled asphalt). Subsequently, after approximately 10 min, the pre-prepared alkaline solution was gradually introduced into the continuously mixing dry blend.

Upon attainment of a homogeneous consistency, the fresh geopolymer mixture was cast into preconditioned molds. The specimens were then subjected to curing in a laboratory oven at 75 °C for 24 h. After demolding, the specimens were stored under laboratory air conditions (20 ± 2 °C, RH ≈ 50%) for 28 days. All tests were conducted after the 28-day conditioning period.

The geopolymerization process was carried out using a 10-molar alkaline sodium hydroxide (NaOH) solution and an aqueous solution of sodium silicate (R-145; molar modulus 2.5; density approximately 1.45 g/cm^3^). These components were combined in a volumetric ratio of 1:2.5 (NaOH:Na_2_SiO_3_).

### 2.3. Examination Methods

#### 2.3.1. X-Ray Diffraction Studies (XRD)

X-ray diffraction (XRD) analyses were conducted using a PANalytical AERIS diffractometer (Malvern Panalytical, Almelo, The Netherlands) equipped with a Cu-Kα radiation source. Measurements were carried out over an angular range of 10° to 100° 2θ, with a scanning increment of 0.003° (2θ). Qualitative phase identification was achieved using the ICDD PDF4+ database. In contrast, quantitative phase analysis was performed using the Rietveld refinement method, which facilitated the determination of the relative proportions of the identified phases.

#### 2.3.2. Chemical Composition Analysis by X-Ray Fluorescence (XRF)

Analysis of the elemental and oxide composition of the materials was performed using an S2 PUMA Series 2 XY Auto-changer X-ray fluorescence (XRF) spectrometer (Bruker AXS SE, Karlsruhe, Germany). Measurements were performed in an air atmosphere.

For the raw materials (fly ash, sand, and asphalt), test samples were prepared as loosely packed powders placed in specialized containers with a base consisting of 4 μm thick polypropylene foil.

For the cured geopolymer samples, measurements were conducted directly on their solid surfaces, without the requirement for supplementary containers or supports.

Samples prepared in this way enabled both qualitative and quantitative analysis of the chemical composition, including both elemental and oxide components, necessary to assess the effect of individual components on the properties of geopolymer materials.

#### 2.3.3. Particle Size Distribution

The particle size distribution of the materials used in the study (i.e., fly ash, sand, and milled asphalt) was determined using an Anton Paar GmbH laser diffraction particle analyzer (Anton Paar GmbH, Graz, Austria) in conjunction with Kalliope Professional software (version 2.22.1).

For fly ash, measurements were performed using a wet laser diffraction technique. The procedure involved preparing a suitable sample, introducing the material into an aqueous dispersion, and then analyzing light scattering in a liquid medium.

For sand and milled asphalt, dry measurements were performed using an Anton Paar GmbH laser diffraction particle analyzer (Anton Paar GmbH, Graz, Austria) equipped with a dry dispersion chamber. The parameters for dry analysis were established as follows:

- vibrator operating time: 70%,
- vibrator frequency: 50 Hz,
- air pressure: 2000 mbar.

All measurements (six repetitions for each material) were conducted under standardized conditions, thereby ensuring the acquisition of reliable and comparable granulometric data for the bulk materials under investigation.

#### 2.3.4. Density

The density of the geopolymer samples was determined via the geometric method, which involved determining the geometric dimensions of the specimens (measured using a laboratory caliper with an accuracy of 0.01 mm) and their mass, determined on a RADWAG PS 200/2000.R2 laboratory scale (maximum load: 200/2000 g; readability: 0.001/0.01 g). The results were averaged based on measurements of three samples for each of the analyzed geopolymer composite variants. Standard deviations were calculated for all obtained values and represented as error bars in the corresponding graphs.

#### 2.3.5. Strength Properties

Flexural strength was determined using samples with dimensions of 40 × 40 × 160 mm, cast in specially adapted molds. Tests were conducted in a three-point bending configuration using a Matest 3000 kN universal testing machine (MATEST S.p.A., Arcore/Treviolo, Italy) in accordance with the PN-EN 12390-5:2019-08 standard [40]. The loading rate was 0.05 MPa/s, and the support spacing was set at 150 mm. Measurements were performed on six samples for each type of prepared geopolymer. In addition to the experimental testing, the flexural strength results underwent statistical analysis. A one-way ANOVA was applied with a significance level of *p* < 0.05 to evaluate the differences between groups, followed by Tukey’s post hoc test. The analysis was performed using Microsoft Excel.

Compressive strength measurements were performed on halves of the samples after the three-point bending tests in accordance with the PN-EN 12390-3:2019-07 standard [41]. The sample halves were placed symmetrically (so that their surfaces were parallel and aligned with the axis of the testing machine plates) on the table of a Matest 3000 kN testing machine, using pressure plates with cross-sectional dimensions of 40 × 40 mm. The test was conducted at the same speed as the bending test—0.05 MPa/s. Measurements were performed on six samples for each type of prepared geopolymer. For the compressive strength data, a one-way analysis of variance (ANOVA) was performed to determine the statistical significance of the differences between mixtures. The analysis was performed at a significance level of *p* < 0.05. Calculations were conducted in Microsoft Excel, and the obtained results were compared with the critical *F*-value.

#### 2.3.6. Abrasion Test

To determine abrasion resistance, rectangular samples measuring 71 × 71 × 71 mm were prepared. Tests were conducted on a Bohme disc (MATEST S.p.A., Arcore/Treviolo, Italy) in accordance with the PN-EN 14157:2017-11 standard [42]. Before testing, it was verified that the opposite surfaces of each sample were parallel to each other and that any irregularities had been removed. The test involved placing the test sample on a rotating disc with an abrasive medium placed on the abrasion path. Artificial corundum was used as the abrasive medium at a dosage of 20 g per abrasion cycle. The sample was pressed against the disc with a load of 294 N. According to the procedure described in the standard, 16 rotational cycles were used during the test, with 22 disc revolutions per cycle. After completing one cycle, the rotating disc was cleaned and the sample rotated 90°. Abrasion tests were conducted on three samples for each of the analyzed geopolymer mixtures. Before and after the test, each sample was weighed and measured. Based on the average values obtained, the material’s abrasion class was determined using the values in Table 2.

#### 2.3.7. Water Mass Sorption Capacity

The mass water sorption capacity (water absorption), which is a measure of the material’s ability to absorb water through pores and capillaries, was determined as the ratio of the mass of water absorbed by the sample to its dry mass, according to the following formula:

(1) $$N\left[\%\right]=\frac{\left(G_{w}-G_{d}\right)}{G_{d}}\cdot 100$$

where *N*—water absorption by mass [%], *G_d_*—mass of the dry sample [g], and *G_w_*—mass of the saturated sample [g].

The water absorption test procedure included the following:

1. Initial step—determining the dry mass of the sample (*G_d_*).
2. Partial immersion—the sample was placed in a beaker and then filled with distilled water to half its height.
3. First measurement—after 2 h, the sample was removed from the water, gently dried on the surface with a soft paper towel, and immediately weighed. The obtained mass was recorded as the first measurement (*G_w,2h_*).
4. Complete immersion—after the first mass measurement, the sample was immersed in distilled water so that it was completely immersed.
5. Daily measurement—measurements were taken successively after 24 h of immersion, and then daily for the next 7 days. Each time, the sample was removed from the water, dried of surface water, and weighed as soon as possible after removal.
6. Weekly measurement—after the weekly series of measurements, when the results stabilized, further measurements were taken at seven-day intervals, continuing observations for one month, while maintaining the same water absorption conditions.

The collected data enabled the observation of the dynamics of water absorption by the geopolymer samples over time, allowing for the determination of the saturation level and absorbency of the individual mixtures. For each composition, the measurements were performed on three independent samples, and the average values were reported.

#### 2.3.8. Microscopic Examination

Microstructural studies of the geopolymers were performed on a JEOL JSM-IT200 scanning electron microscope (JEOL, Akishima, Tokyo, Japan). Before SEM imaging, the specimens were dried at 40 °C for 24 h. Prior to observation, the samples were sputter-coated with a thin layer of gold using a vacuum coater (model DII-29030SCTR, JEOL, Akishima, Tokyo, Japan) at an accelerating voltage of 10 kV for 120 s. This preparation enabled the acquisition of high-quality images of the geopolymer microstructure.

## 3. Results and Discussion

### 3.1. Material Characteristics

#### 3.1.1. Fly Ash

**Figure 1:**
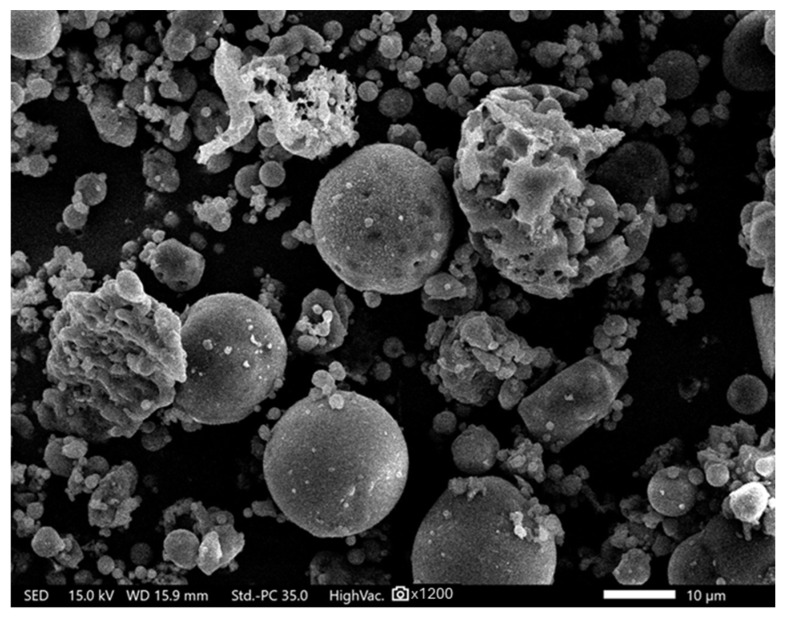
SEM image of fly ash; ×1200 magnification.

Figure 1 presents a scanning electron microscopy (SEM) image of raw fly ash. SEM analysis of the fly ash revealed a typical spherical morphology, characteristic of class F fly ash. The smooth surfaces of the cenospheres improve the workability of the mixtures, while the presence of porous and fractured particles increases the material’s reactivity in geopolymerization processes. Such morphological diversity may influence both the rheological and mechanical properties of the geopolymer composites. Fragments of irregular particles are also visible, resulting from the spheres cracking during rapid cooling following coal combustion.

**Figure 2:**
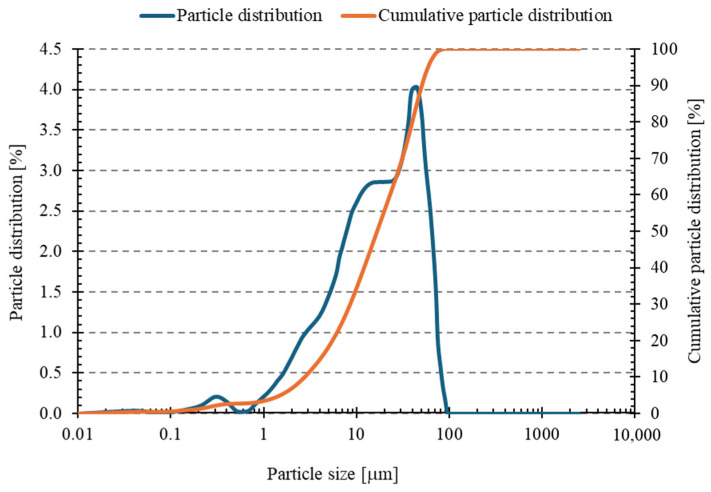
Particle size distribution curve and cumulative curve of fly ash.

Figure 2 displays the results of the analysis of fly ash particle size distribution analysis (measured using the wet method). Table 3 summarizes statistical data obtained from six measurements of the ash particle size distribution, including the following diameter values: D10, representing the mean diameter of particles with a share not exceeding 10 percent of the tested sample; D50 median, which is the mean diameter of particles with a share not exceeding 50 percent of the tested sample; and D90, which is the mean diameter of particles with a share not exceeding 90 percent of the tested sample. Additionally, Table 3 also provides the dispersion index value.

Particle size distribution analysis (Figure 2 and Table 3) revealed that the fly ash used in the study was a fine-grained material, with a mean particle size of 23.59 μm (SD = 0.25 μm). The median value of D_50_ was 16.53 μm, while D_10_ and D_90_ were 2.71 μm and 50.79 μm, respectively. The dispersion index (Span) of 2.91 indicates a relatively wide particle size distribution, with low standard deviations for all parameters, confirming high measurement repeatability. The dispersion index of 2.908 indicates a relatively wide particle size distribution in the tested sample, indicating that the particles have a wide range of sizes. However, the standard deviations for individual parameters are relatively low: from 0.047 to 0.946 µm, indicating the repeatability of the measurements and the stability of the results. The relative standard deviations expressed as percentages are also very low, further confirming the high precision of the measurements.

**Figure 3:**
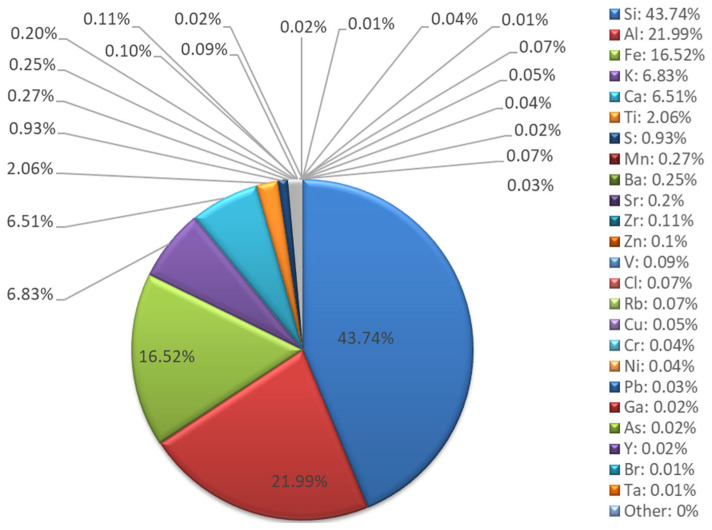
Fly ash XRF analysis results—elemental composition.

**Figure 4:**
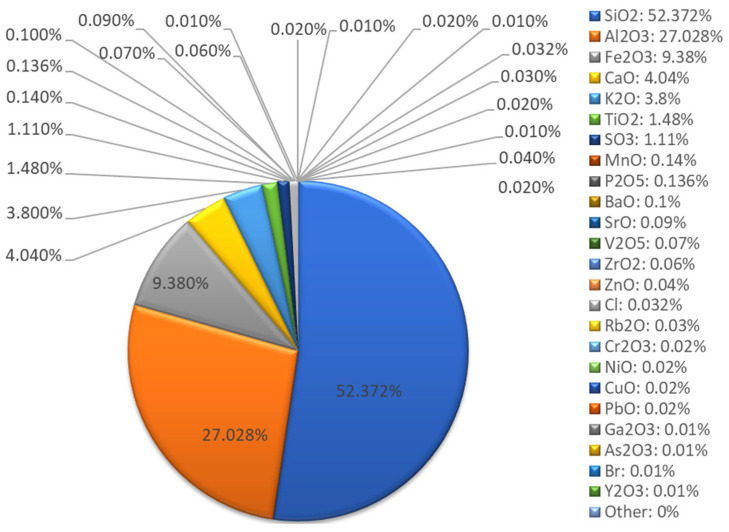
Fly ash XRF analysis results—oxide composition.

XRF chemical composition analysis (Figure 3 and Figure 4) confirmed that the fly ash belongs to class F, according to the ASTM C618 classification [44]. The dominant elemental composition is silicon (43.74%), aluminum (21.99%), iron (16.52%), and calcium (6.51%). The high Si and Al content translates into a favorable Si:Al molar ratio of 1.99, favoring the formation of a stable geopolymer network [45]. The presence of potassium (6.83%) may additionally support the alkaline activation process. Trace elements, including Ni, Cu, Zn, Pb, and As, are present at low concentrations (0.01–0.1%), indicating minimal environmental contamination and supporting the suitability of the fly ash for construction applications.

The oxide composition indicates a predominance of SiO_2_ (52.37%), Al_2_O_3_ (27.03%), Fe_2_O_3_ (9.38%), and CaO (4.04%), which aligns with the typical range observed for low-calcium fly ash utilized in geopolymer synthesis [39].

**Figure 5:**
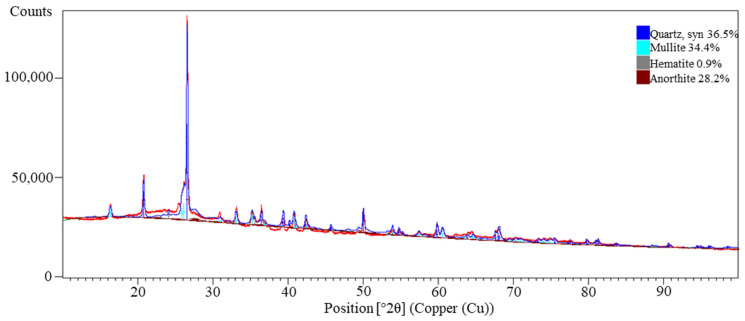
X-ray diffraction (XRD) pattern of the investigated fly ash showing the identified crystalline phases; XRD pattern (red), background (green).

Additionally, a qualitative phase analysis was performed using X-ray diffraction (XRD) (Figure 5). The diffraction spectrum revealed the presence of four main crystalline phases: quartz (36.5%; card No.: 01-083-0539), mullite (34.4%; card No.: 04-027-3775), anorthite (28.2%; card No.: 04-015-4988), and hematite (0.9%; card No.: 04-008-8479). Quartz and mullite are characteristic constituents of coal-derived fly ash, formed as a result of high-temperature coal combustion, which is consistent with the observations of other authors [46,47]. The notable presence of anorthite indicates a significant fraction of reactive calcium aluminosilicates, which may influence the microstructure and mechanical attributes of the resulting geopolymers [48]. The low hematite content is typical and should not significantly impact the final properties of the material, although it may affect its color and, to some extent, its radiation absorption [49,50]. The high content of amorphous phases (not directly visible in XRD analysis but confirmed by XRF results, as evidenced by high Si and Al content) suggests significant reactivity of the tested fly ash. According to the literature, amorphous aluminosilicates are the primary source of reactive precursors in the geopolymerization process, influencing both the reaction kinetics and the final strength of the material [1].

#### 3.1.2. Sand

**Figure 6:**
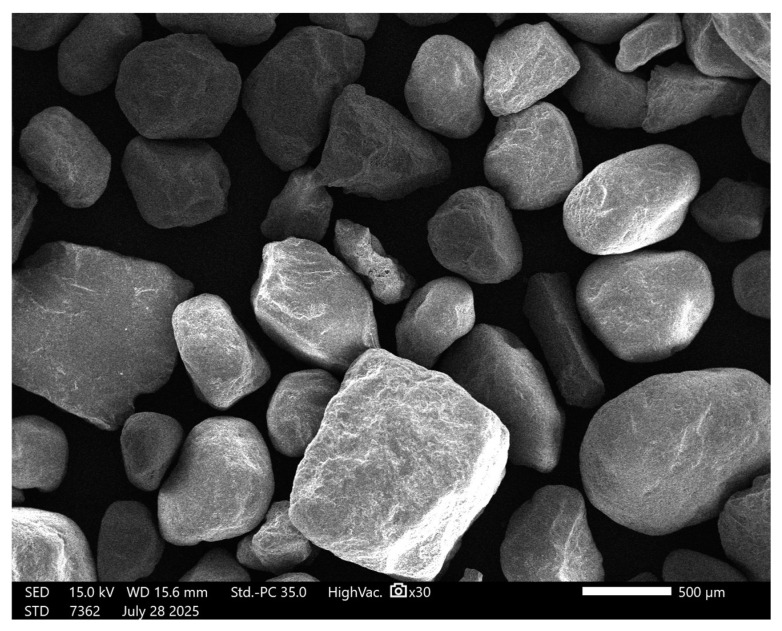
SEM image of sand; ×30 magnification.

The morphology of sand grains (Figure 6) is characterized by irregular shapes and sharp edges, which favor mechanical anchoring in the geopolymer matrix. The presence of micro-irregularities on the surface can increase the contact area with the binding phase, but the lack of fines limits the ability to fill the micropores.

**Figure 7:**
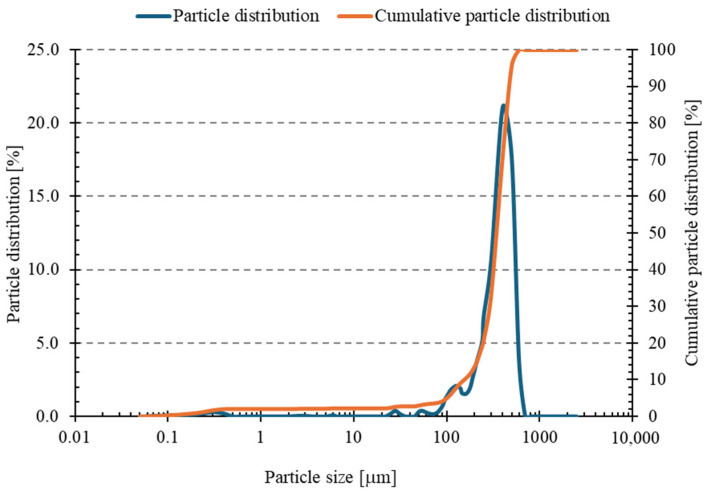
Particle size distribution curve and cumulative curve of sand.

Analysis of the sand particle size distribution (Figure 7 and Table 4) revealed that the material was coarse-grained, with a mean particle size of 374.15 μm (standard deviation, SD = 5.92 μm). The D_10_, D_50_, and D_90_ values were 182.57 μm, 341.72 μm, and 474.50 μm, respectively. The dispersion index (Span) of 0.854 indicates a relatively narrow particle size distribution. Analysis of standard deviations demonstrated high measurement repeatability, with the most significant variation in the fine fraction (D_10_). On this basis, it can be concluded that the sand used in the tests exhibits relatively good granulometric homogeneity, which is beneficial for achieving a uniform structure of the geopolymer mass. Smaller particle dispersion promotes better compaction and homogeneity of the material, which may translate into improved mechanical properties and durability of the final product [51].

**Figure 8:**
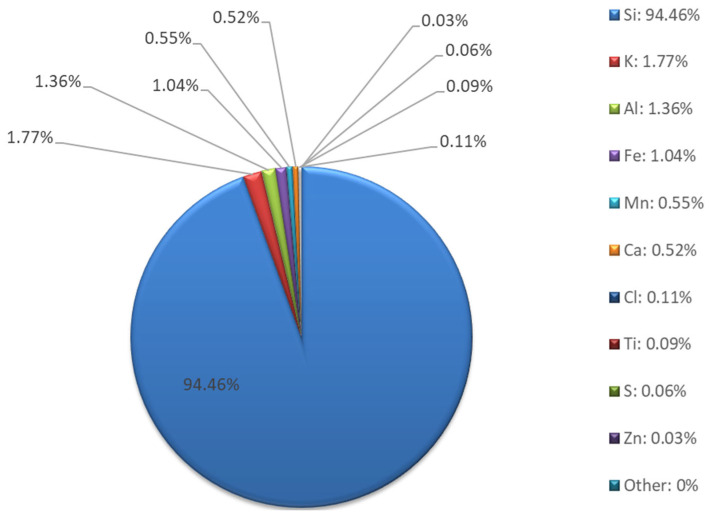
Sand XRF analysis results—elemental composition.

**Figure 9:**
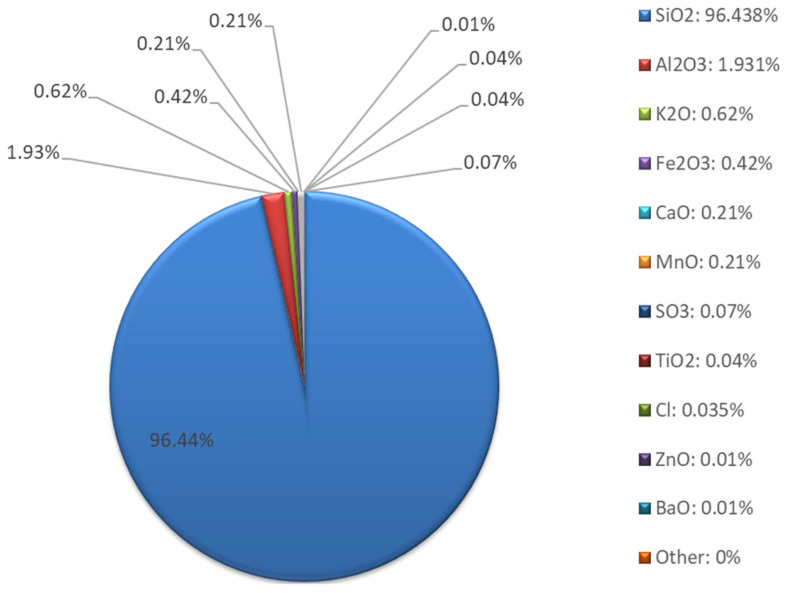
Sand XRF analysis results—oxide composition.

Chemical composition studies using XRF (Figure 8 and Figure 9) confirmed that the sand is typically composed of silica. The elemental composition is dominated by silicon (94.46%), with low contents of aluminum, potassium, calcium, and iron. The presence of small amounts of Al, K, Ca, and Fe may indicate the presence of clayey or sedimentary rocks and naturally occurring oxide compounds. The absence of sodium, magnesium, and most heavy metals (Pb, Ni, Cu, Zn, Cr, As, etc.) indicates very low environmental contamination and a high level of material purity.

The oxide composition revealed that the main component was SiO_2_ (96.44%), with small amounts of CaO, K_2_O, and Fe_2_O_3_. The very low SO_3_ (0.07%) and Cl_3_ (0.035%) contents indicate the absence of significant organic contaminants or industrial residues.

**Figure 10:**
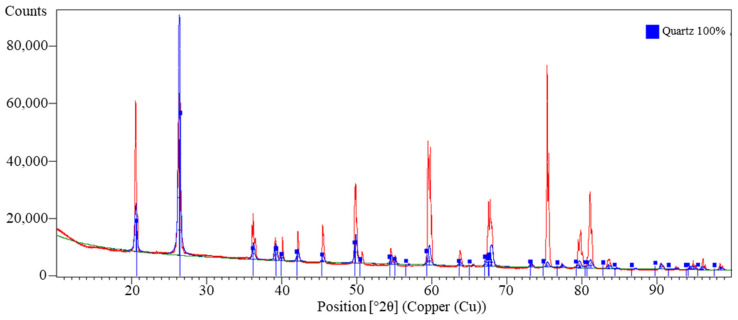
X-ray diffraction (XRD) pattern of the investigated sand showing the identified crystalline phases: XRD pattern (red), background (green).

XRD phase analysis (Figure 10) showed that the tested sand consists almost entirely of quartz (100%; card No.: 01-074-1811). The absence of other crystalline phases indicates high mineralogical purity. Highly crystalline quartz is practically insoluble under geopolymerization conditions and acts mainly as an inert filler that improves dimensional stability and abrasion resistance [52,53]. Its influence on chemical processes is limited, but thanks to the “skeletal” effect of the particles, it can support structural densification [54].

#### 3.1.3. Asphalt from Road Demolition

**Figure 11:**
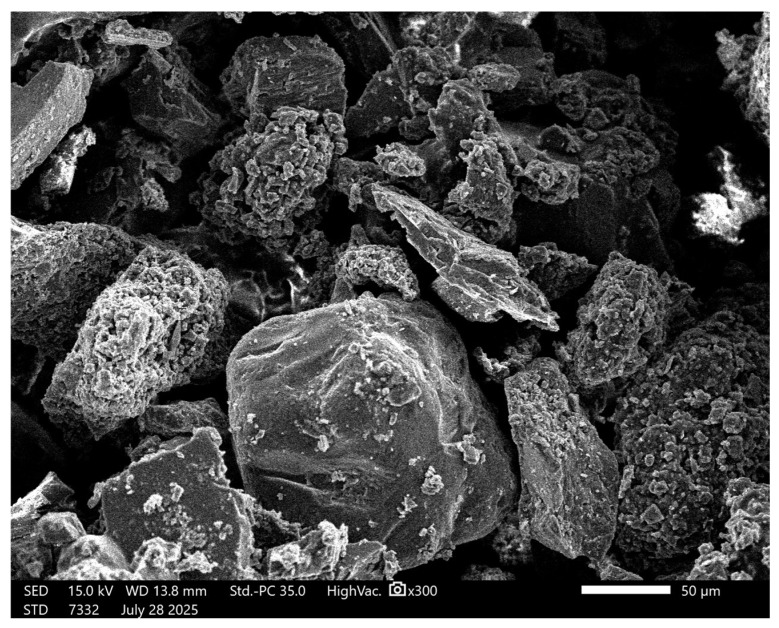
SEM image of asphalt.

The structure of milled asphalt (Figure 11) includes irregular, angular particles with a highly developed cracked surface (which promotes mechanical bonding in the composite), with visible zones of detachment of the asphalt binder from the mineral aggregate.

**Figure 12:**
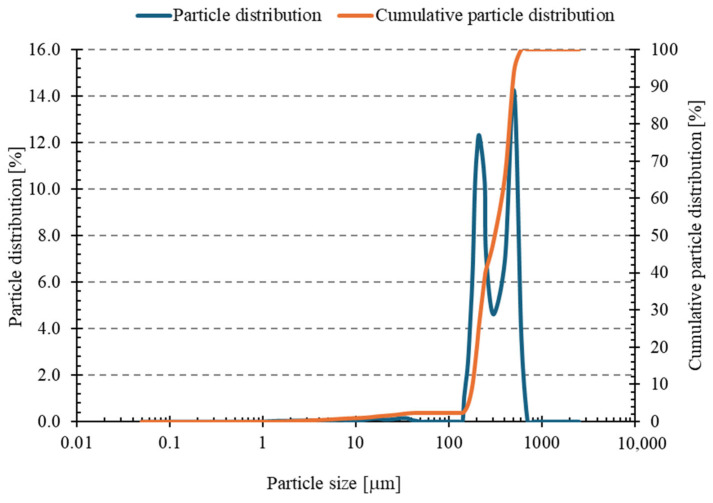
Particle size distribution curve and cumulative curve of asphalt.

Analysis of the asphalt particle size distribution after crushing and sieving (Figure 12 and Table 5) revealed that it was a coarse-grained material with a mean particle size of 311.54 μm (standard deviation, SD = 88.08 μm). The D_10_, D_50_, and D_90_ values were 172.95 μm, 250.58 μm, and 437.08 μm, respectively. The high standard deviation and relative percentage deviation (28.27%) indicate significant grain heterogeneity. The dispersion index (Span) of 1.177 confirms the broad particle size distribution, which may negatively impact the homogeneity of the geopolymer mass and lead to local structural discontinuities [55].

**Figure 13:**
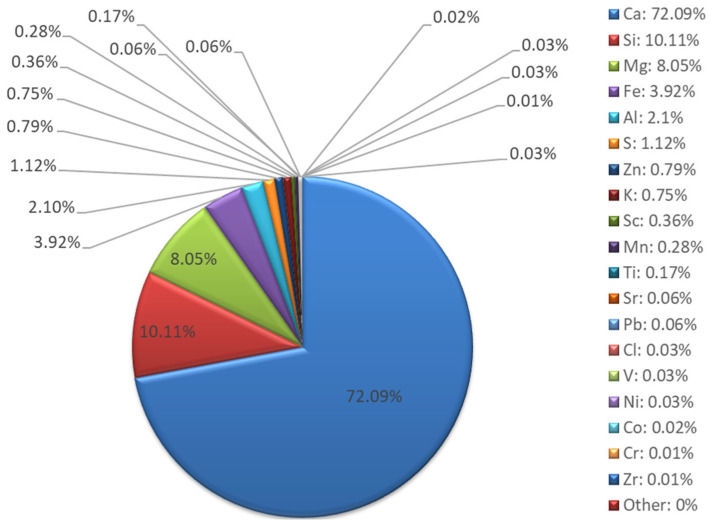
Asphalt XRF analysis results—elemental composition.

**Figure 14:**
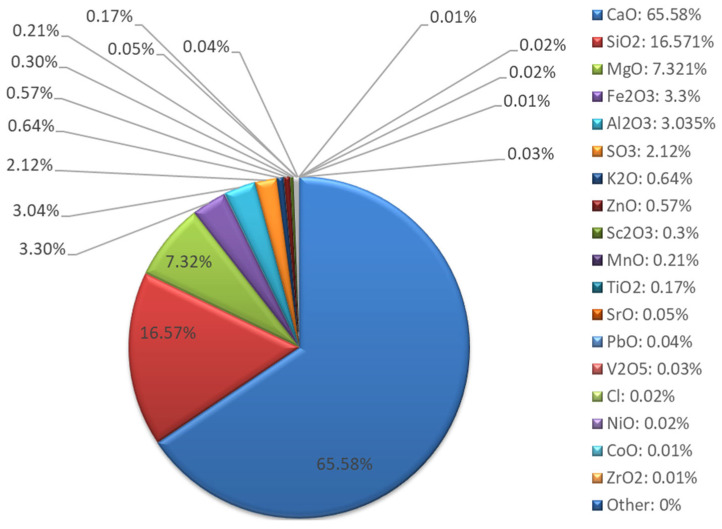
Asphalt XRF analysis results—oxide composition.

Chemical composition studies using the XRF method (Figure 13 and Figure 14) showed that milled recycled asphalt is characterized by a dominant calcium content (72.09%), originating mainly from calcium carbonates (CaCO_3_) and potential cement residues. Mg (8.05%), Si (10.11%), Al (2.10%), Fe (3.92%), K (0.75%), and S (1.12%) are also present. Such proportions indicate a high share of mineral admixtures, including limestone grits and anti-slip additives. The low Si and Al content compared to fly ash limits the reactivity of asphalt as a precursor material, making it rather a filler or modifying additive in geopolymer mixtures [56].

**Figure 15:**
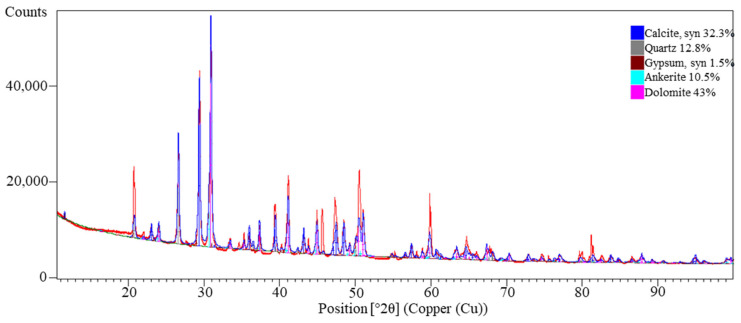
X-ray diffraction (XRD) pattern of the investigated asphalt showing the identified crystalline phases; XRD pattern (red), background (green).

XRD phase analysis (Figure 15) revealed the presence of five main crystalline phases: dolomite (43.0%; card No.: 04-023-8809), calcite (32.3%; card No.: 04-008-0788), quartz (12.8%; card No.: 01-070-3755), ankerite (10.5%; card No.: 00-041-0586), and gypsum (1.5%; card No.: 04-016-3025). Dolomite and calcite dominate the mineral composition, confirming the XRF results, which indicate a high proportion of Ca and Mg. The presence of quartz results from mineral admixtures in the asphalt mixture, while gypsum may originate from impurities or sulfate admixtures used in road construction [57,58].

Such mineralogical and chemical composition causes milled asphalt to act mainly as a neutral aggregate in geopolymer mixtures, which can improve abrasion resistance and dimensional stability, but is not a significant source of reactive aluminosilicates [1].

### 3.2. Density Measurement Results

Bulk density is one of the fundamental physical parameters of building materials. It influences both mechanical strength and functional properties. In the case of geopolymer composites, the density value can indicate the effectiveness of the mixture’s compaction and the presence of porosity within the material.

**Figure 16:**
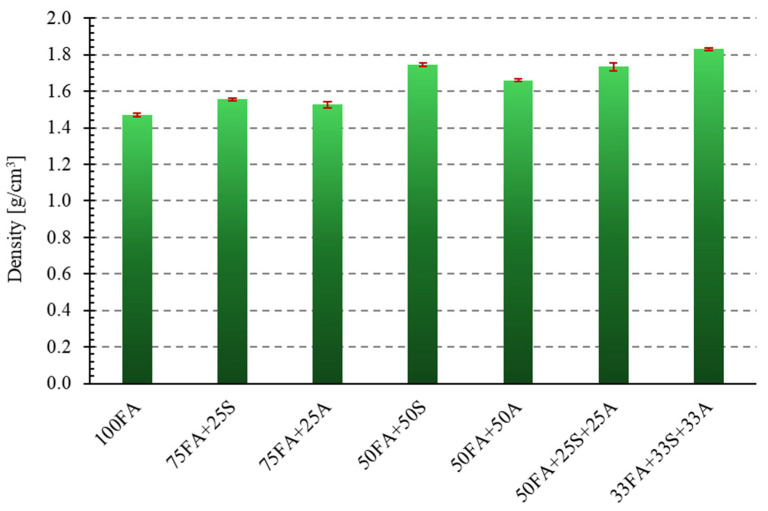
Density of analyzed geopolymer samples.

This study analyzed the density of samples made from class F fly ash with the addition of sand and milled asphalt in various proportions. A composite made solely from fly ash (designation: 100FA) was adopted as the reference sample. Density measurements for all manufactured geopolymer samples were performed using the geometric method. Figure 16 graphically presents the obtained density measurement results for all samples compared to the reference sample 100FA for the samples modified with sand and asphalt, respectively.

Density analysis revealed a significant effect of the type and amount of additives used on the bulk properties of the tested geopolymers. The reference sample, made of 100% fly ash (100FA), had a density of 1.464 g/cm^3^. Adding 25% sand (75FA + 25S) increased the density by approximately 6.3%, while increasing its content to 50% (50FA + 50S) resulted in a 19.5% increase in density. An even more pronounced effect was observed in the case of three-component mixtures—the 33FA + 33S + 33A variant reached a value of 1.839 g/cm^3^, representing a 25.5% increase compared to the baseline sample.

The obtained results are consistent with the observations of Gao et al. [59], Tracz et al. [60], and Zawrah et al. [61] according to which the increase in geopolymers’ density is directly related to the filling of voids in the structure and the reduction in porosity due to the presence of complex, mineral fractions with appropriate grain size. In the case of sand, its high density and relatively good compatibility with the aluminosilicate matrix promote densification of the system. However, the presence of asphalt—an organic material with lower density and weaker adhesion to the geopolymer gel—not only does not improve this parameter but can also increase the proportion of closed pores, which explains the lack of a significant increase, and in one case, even a slight decrease, compared to the base sample.

A similar effect of the limited influence of asphalt waste on the density of geopolymers was also noted by Liu et al. [19], indicating that the key factor in improving this parameter is the presence of mineral filler fractions, not the mass fraction of the additive itself. In practice, this means that for applications requiring high density and the associated greater strength (e.g., prefabricated structural elements, paving slabs, paving stones), the optimal solution is to use sand as the main additive. At the same time, asphalt materials can only be introduced in limited quantities or in combination with mineral fillers.

### 3.3. Strength Test Results

#### 3.3.1. Flexural Strength

Flexural strength is a crucial mechanical parameter, particularly for brittle materials like geopolymers, which may be subjected to tensile stresses in real-world conditions. In this study, three-point bending measurements were performed to determine the maximum flexural stress (MPa) for all tested materials, which differed in composition. Each measurement was performed on a series of six samples, and then the mean values and standard deviations were determined. The results of the one-way ANOVA (Table 6) confirmed that the differences in flexural strength between the tested compositions were statistically significant (F = 35.90, *p* < 0.0001). This indicates that the addition of both quartz sand and milled asphalt had a substantial impact on the flexural strength of the geopolymer composites.

**Figure 17:**
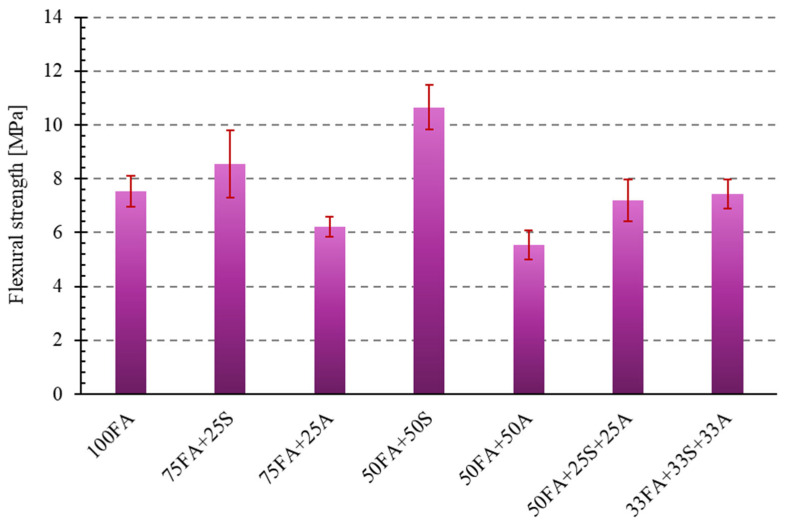
Flexural strength of the tested geopolymer compositions.

Figure 17 presents a bar graph representing the obtained flexural strength results for all tested geopolymer compositions.

Analysis of the obtained results revealed a significant variation in the strength of the samples depending on the additive used. The use of sand as a mineral component had a positive effect on the material’s flexural strength. The addition of 25% sand (75FA + 25S) achieved a strength of 8.54 MPa, representing a 13.3% increase compared to the reference sample. Even better results were recorded for the sample with 50% sand (50FA + 50S), where the average strength was 10.65 MPa, a 41.2% increase compared to the 100FA sample. Based on the obtained results, it can be assumed that the fine-grained mineral structure of sand favors the formation of a compact and homogeneous geopolymer matrix, thereby increasing its mechanical resistance.

In contrast to the effects obtained with sand as an additive, the use of milled asphalt had a negative impact on the mechanical properties of the samples. In the case of sample 75FA + 25A, the strength decreased by 17.5%, and in the sample with 50% asphalt (50FA + 50A), by as much as 26.5% compared to the reference. This decrease may be related to the irregular structure of the asphalt particles, the presence of organic fractions, and potential poor interfacial adhesion in the geopolymer matrix. In the following steps, selecting a smaller, more homogeneous asphalt fraction should be considered.

Interesting results were obtained for the ternary composite materials, containing both sand and asphalt. Sample 50FA + 25S + 25A achieved a strength of 7.19 MPa (a 4.6% decrease), while sample 33FA + 33S + 33A reached 7.42 MPa, which is only 1.6% less than the reference sample. The results indicate that the simultaneous use of a mineral and waste additive can partially offset the negative impact of using only asphalt. A properly selected proportion of ingredients can allow for the use of recycled materials without significant loss of mechanical properties.

Similar studies have shown that appropriately selected mineral additives significantly increase flexural strength by reducing micro-crack initiation [38]. The presence of asphalt weakens the structure, but when combined with sand, its negative impact is partially neutralized. In the context of geopolymer applications, sand-based versions are beneficial for elements subjected to bending, such as paving slabs or thin-walled precast elements.

#### 3.3.2. Compressive Strength

One of the most essential mechanical parameters determined for building materials is compressive strength. A sufficiently high compressive strength ensures the durability and operational safety of materials for engineering applications.

**Figure 18:**
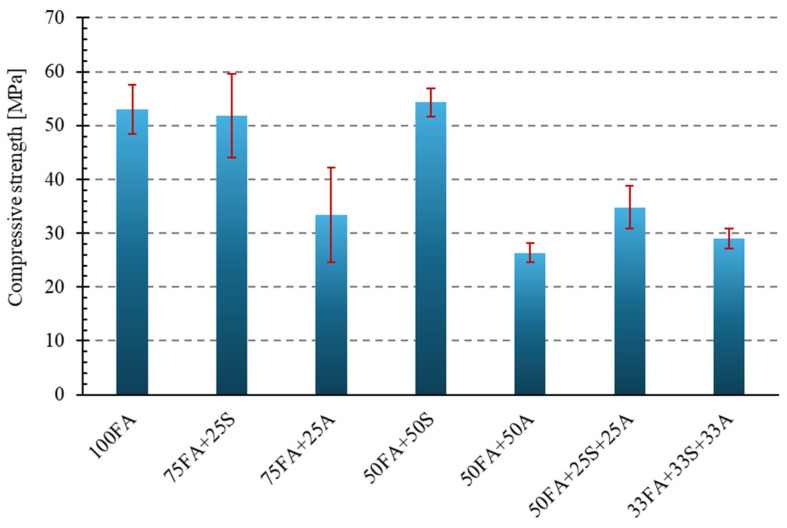
Compressive strength of the tested geopolymer compositions.

Figure 18 presents a bar graph showing the compressive strength results for all tested materials.

The addition of 25% sand (sample 75FA + 25S) resulted in a slight decrease in strength to 51.83 MPa, representing a 2.2% difference compared to the reference sample. Although the relatively high standard deviation caused the mean values to visually overlap with those of the reference sample, the ANOVA analysis confirmed that the differences between the groups are statistically significant (*p* < 0.0001). The sample containing 50% sand (50FA + 50S) had a slightly higher average strength of 54.26 MPa, representing a 2.4% increase compared to the sample containing fly ash without additives.

A much more noticeable effect on compressive strength was observed for the samples with milled asphalt. The sample with 25% asphalt (75FA + 25A) showed an average compressive strength of 33.39 MPa, representing a 37.0% decrease compared to the reference sample. An even more unfavorable result was obtained for sample 50FA + 50A, whose average strength was only 26.36 MPa—a decrease of over 50% compared to the sample made with fly ash alone. This significant decrease is most likely due to the heterogeneous structure of the asphalt additive, as well as the presence of organic components that do not participate in the bonding process and can further disrupt the internal structure of the composite. It is also possible that the asphalt causes increased porosity or the appearance of weakened zones in the geopolymer matrix, which in turn leads to sample failure under lower load values.

For the three-component samples, containing both sand and asphalt, intermediate strength values were obtained. Sample 50FA + 25S + 25A achieved a result of 34.83 MPa (a decrease of 34.3%), while sample 33FA + 33S + 33A achieved a result of 29.03 MPa (a reduction of 45.2%). Although the compressive strength values obtained in these cases are significantly lower than for the base sample, it is worth noting that the addition of sand partially compensates for the negative impact of asphalt. This means that it is possible to use recycled raw materials—even those of lower technical value, such as asphalt—without completely losing the material’s functionality, provided their share is appropriately balanced by the mineral component. Therefore, mixtures containing both sand and asphalt, which exhibit limited compressive strength, could be used for less stressed components.

The statistical evaluation of compressive strength results, using one-way ANOVA (Table 7), showed a highly significant effect of the mixture composition on mechanical performance (F = 28.83, *p* < 0.0001). This confirms that the addition of sand or asphalt modifies the load-bearing capacity of the composites in a non-random manner. The geopolymer matrix remains the primary binding phase, but its integrity and load transfer capacity can be either improved or disrupted, depending on the type and proportion of the additive.

The literature suggests that mineral fractions, such as sand, yield more stable geopolymer structures due to improved adhesion and reduced porosity. In turn, organic components without binding properties, such as asphalt, reduce matrix cohesion—a similar effect was noted in studies on asphalt recycling in PMC [38]. The results indicate that geopolymers with a high asphalt content can be used for low mechanical requirements (e.g., as base layers). In contrast, those with a high sand content are suitable for structural applications.

### 3.4. Abrasion Test Results

**Figure 19:**
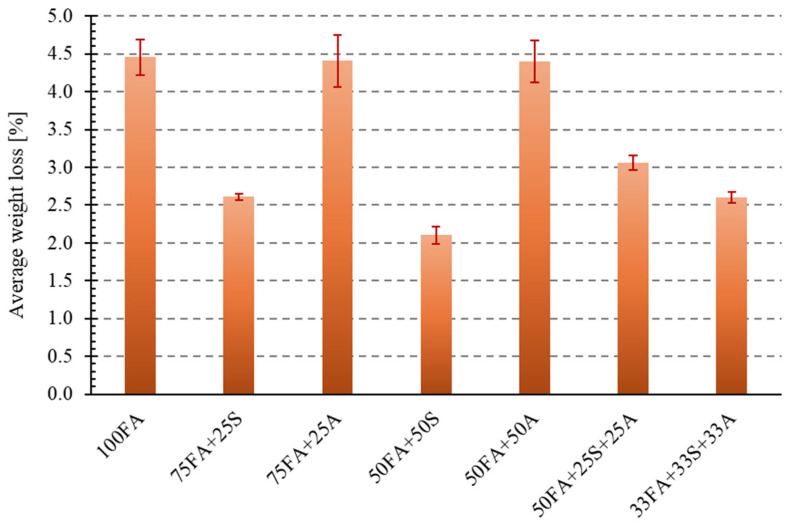
Abrasion resistance test results—average weight loss.

**Figure 20:**
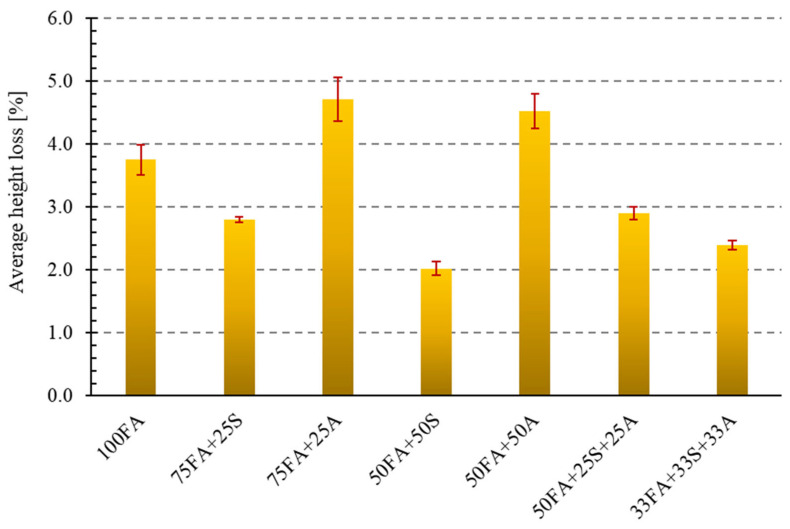
Abrasion resistance test results—average height loss.

Another essential characteristic that building materials such as industrial flooring, paving stones, curbs, and road elements must possess is abrasion resistance. The study analyzed the abrasion resistance of the geopolymer mixtures tested using a Bohme disk (Matest, Treviolo, Italy). The assessment was based on the average mass loss and average height loss of the samples after the abrasion tests. Based on the height losses, the samples were assigned appropriate abrasion classes according to the adopted criteria presented in the methodology. The results are presented in Figure 19 and Figure 20.

The obtained test results indicate significant differences in abrasion resistance between the individual sample types, depending on the material additives used. The most considerable mass losses were recorded for the reference sample (4.46%) and the samples with 25% and 50% asphalt additive, which had mass losses of 4.41% and 4.40%, respectively. The lowest mass losses were recorded for the 50FA + 50S sample (2.10%), suggesting a positive effect of this additive on increased cohesion and, consequently, abrasion resistance. Similarly, good results were achieved for the ternary composite materials (33FA + 33S + 33A, 2.60%, and 50FA + 25S + 25A, 3.06%), indicating a beneficial synergistic effect of the various additives.

Analyzing the percentage values of height loss for the tested samples revealed a correlation with the mass loss measurements. The reference sample made solely of fly ash (100FA) achieved an average height loss of 3.75 mm, qualifying it as a low-abrasion material. The addition of sand, both at 25% (75FA + 25S) and 50% (50FA + 50S), significantly improved the samples’ resistance to abrasive forces. In these cases, height losses were 2.80 mm and 2.03 mm, respectively, classifying both material groups as very low-abrasion materials. The reduction in height loss for the 50FA + 50S material by over 45% compared to the reference sample may be due to the higher hardness of the mineral additive, which limits the frictional wear of the composite structure.

The opposite trend was observed for the addition of milled asphalt. The most significant height loss occurred in the case of the 50FA + 25A material, confirming the previously observed trend that the presence of milled asphalt, especially in high concentrations, can negatively impact abrasion resistance. Samples containing 25% and 50% asphalt exhibited significantly higher height losses—4.71 mm and 4.52 mm, respectively—indicating that they are classified as low-abrasion materials. It can be assumed that the presence of the organic component in the form of asphalt likely weakens the material’s surface resistance, resulting in the formation of microcracks and increased erosion upon mechanical contact.

The results obtained for the three-component samples, similar to the mass loss analysis, yielded intermediate results. Sample 50FA + 25S + 25A achieved an average height loss of 2.90 mm, while 33FA + 33S + 33A achieved an average height loss of 2.40 mm. The obtained results allow these materials to be classified as low and very low abrasion, respectively. Of particular interest is the result for sample 33FA + 33S + 33A, which, with a balanced composition, achieved a significantly better outcome than the sample made with pure ash.

Literature indicates that a surface containing hard fractions, such as sand, significantly increases the abrasion resistance of PMC geopolymers [62,63,64]. This study confirmed this effect. Both composites containing only sand and composites containing sand and asphalt from road demolition in appropriate proportions are ideal for situations where surface hardness is crucial (industrial floors, road verges, and paving stones).

### 3.5. Results of Water Mass Sorption Capacity Tests

**Figure 21:**
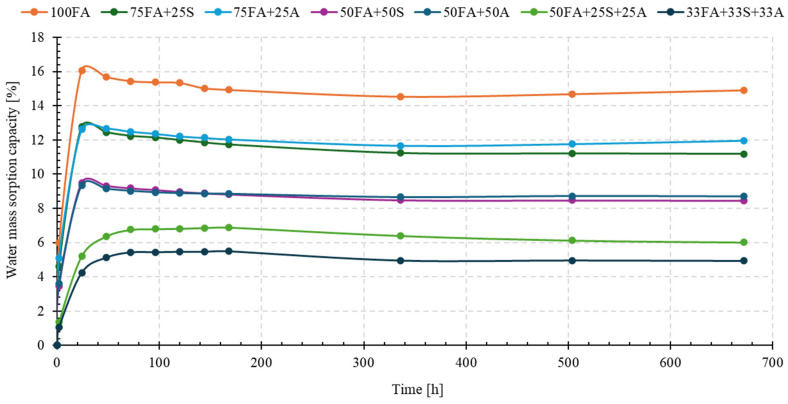
Mass water absorption capacity of the tested geopolymer materials.

The ability of geopolymers to absorb water is one of the key parameters determining their durability, resistance to atmospheric factors, and susceptibility to degradation in environments with variable humidity [65,66]. High water absorption promotes the initiation of frost processes and alkaline corrosion and can also lead to a reduction in mechanical parameters during operation. Water absorption testing was conducted for 28 days, recording the change in sample mass over time. The results are presented in Figure 21.

In the initial phase (the first dozen or so hours), a rapid mass increase was observed, which is typical of capillary water absorption into the material’s porous structure. After this stage, the absorption rate slowed significantly, and the water absorption curves reached a quasi-equilibrium state.

The highest water absorption was recorded for the 100FA reference sample, exceeding 16%, indicating high open porosity and a large capillary volume within the structure. Similar levels (12–13%) were observed for the 75FA + 25S and 75FA + 25A mixtures. The addition of sand or asphalt in these proportions partially sealed the structure, but this effect was insufficient to significantly reduce the sorption capacity. These results correspond to the observations of Mehmani et al. [67] and Brantut et al. [68], who demonstrated that a small proportion of mineral fraction only reduced microporosity, leaving the open capillary network largely intact.

Significantly better water absorption parameters were obtained for the 50FA + 50S (approx. 9%), 50FA + 25S + 25A (approx. 6.5%), and 33FA + 33S + 33A (below 4%) mixtures. In particular, the three-component variant demonstrated the lowest sorption capacity, indicating a compact and low-porosity structure. This phenomenon can be attributed to the synergistic effect of mineral and organic components, which limit both microporosity and capillary pore diameter [69].

An interesting observation is the result for 50FA + 50A (approx. 5%), which, despite the significant asphalt content, was characterized by relatively low water absorption. The reduced water absorption observed in mixtures with a high content of asphalt should not be explained exclusively by the hydrophobic nature of the additive [37,38,70]. The effect is also likely connected with the formation of a heterogeneous pore system, where closed pores and weak interfacial zones limit the penetration and migration of water. Therefore, the low sorptivity may result from the combined influence of both mechanisms.

The obtained results have significant implications for the application. Materials with high water absorption, such as 100FA, should be used primarily in environments with low moisture exposure. However, compositions with water absorption below 5% (e.g., 50FA + 50A, 33FA + 33S + 33A) can be recommended for use in outdoor infrastructure, including elements exposed to freeze–thaw cycles and periodic exposure to rainwater [71,72].

### 3.6. Chemical Composition of the Tested Geopolymer Composites

X-ray fluorescence (XRF) analysis allowed us to determine the elemental and oxide composition of geopolymers with varying amounts of fly ash, quartz sand, and milled asphalt. The results are summarized in Table 8 and Table 9, enabling us to track changes in the content of the main mineral components and assess the impact of individual additives on the final material composition.

In all tested samples, the dominant elements were silicon (Si), aluminum (Al), iron (Fe), potassium (K), and calcium (Ca), which is characteristic of composites based on fly ash and mineral aggregates [1]. The highest silicon content was recorded in the 50FA + 50S sample (49.37%), which is attributed to the presence of a significant amount of quartz sand (SiO_2_). High Si values were also recorded in the reference sample 100FA (48.22%) and in the 50FA + 50A mixture (48.01%), confirming that fly ash provides significant amounts of amorphous silica. In ternary composite materials, the Si content was lower—in the 50FA + 25S + 25A mixture, it reached a minimum of 42.74%. Aluminum content showed a decreasing trend with decreasing FA content—from 13.81% in the reference sample to 9.41% in the 50FA + 25S + 25A composite. This element, originating primarily from the aluminosilicate phase of fly ash, plays a crucial role in geopolymerization processes [1,2]. Reducing its content can lead to a decrease in the degree of polycondensation of the geopolymer gel, and thus to a reduction in mechanical strength.

For iron, the highest values were recorded in the 75FA + 25A sample (19.63%), which may be related to the presence of Fe in both the fly ash and the road asphalt. The addition of sand, on the other hand, promoted an increase in magnesium and calcium content—particularly in the 75FA + 25S mixture (Mg = 4.39%, Ca = 7.58%). The highest Ca content was found in sample 33FA + 33S + 33A (9.58%), suggesting the presence of carbonates and road cement residues in the sand and asphalt [37].

High sulfur content was found in samples 50FA + 25S + 25A (11.09%) and 50FA + 50S (3.89%). The source of S may be sulfate residues in the asphalt and industrial fractions in the sand. An increased SO_3_ content may negatively impact the material’s resistance to aggressive environments [1].

Oxide analysis revealed the dominance of SiO_2_ (53.69–61.26%) and Al_2_O_3_ (11.13–16.72%) in all tested blends. Changes in the share of these oxides were closely correlated with the FA and S proportions, which is consistent with the observations of other authors [1,73,74,75]. The CaO content ranged from 4.54% (50FA + 50S) to 6.19% (33FA + 33S + 33A), which may favor the formation of secondary C–A–S–H phases, beneficial for mechanical strength but increasing susceptibility to carbonation. The content of Fe_2_O_3_ was stable (9.83–12.06%), and the remaining components, such as K_2_O (4.0–4.4%), TiO_2_ (~1.5%), or MnO (~0.2%), showed slight fluctuations and are typical for fly ash. The contents of trace oxides (CuO, ZnO, As_2_O_3_, V_2_O_5_, Rb_2_O) remained at the level of <0.1% and did not play a significant role in shaping the properties of the composites.

In addition to specifying the elemental and oxide composition, the XRF analysis results enable the calculation of the Si/Al ratio, a key parameter that controls the structure and performance of geopolymers. In the case of the 100FA reference sample, the Si/Al ratio was relatively balanced, corresponding to satisfactory strength. Mixtures with a higher quartz sand content exhibited a higher Si/Al ratio, resulting in improved strength properties. This is because a moderate increase in the Si/Al ratio promotes the formation of a more cross-linked and dense geopolymer network. However, asphalt-modified mixtures exhibited a lower effective Si/Al ratio due to the presence of non-reactive phases, which ultimately resulted in reduced flexural and compressive strength. The obtained results confirm that the balance between reactive silica and alumina has a strong influence on mechanical properties, while non-reactive asphalt disrupts this balance. Furthermore, the presence of CaO, especially in ternary composite materials (up to 6.19% in 33FA + 33S + 33A), may further promote the formation of secondary C–A–S–H phases, providing a partial increase in strength. Therefore, the observed mechanical trends can be directly related to the compositional changes revealed by XRF analysis.

### 3.7. Microscopic Examination Results

**Figure 22:**
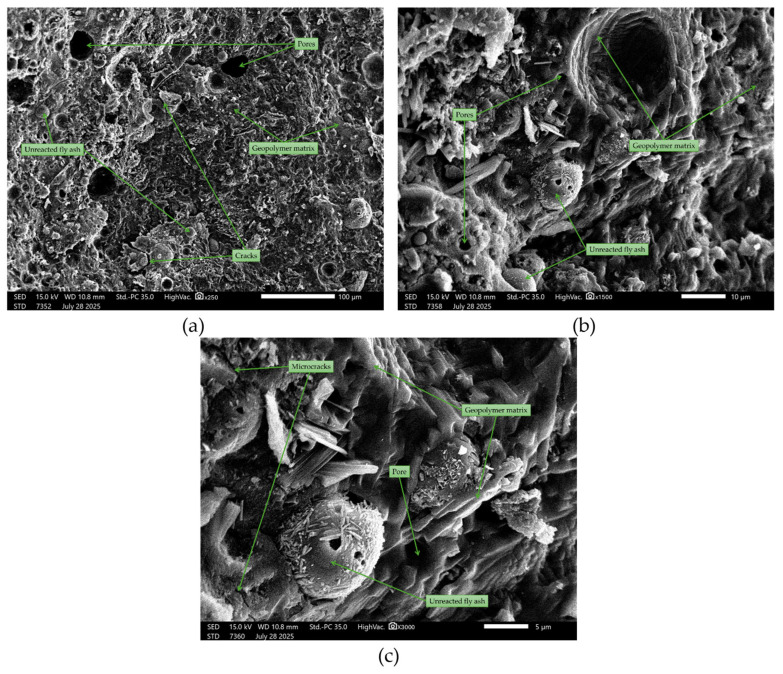
SEM images of the reference geopolymer (100FA): (**a**) ×250 magnification; (**b**) ×1500 magnification; (**c**) ×3000 magnification.

Microstructural studies were performed using a scanning electron microscope (SEM) in secondary electron detector (SE) mode. A reference sample and samples from both the binary (sand/asphalt with geopolymer binder) and ternary (sand, asphalt, and geopolymer binder) composite materials were analyzed. SEM observations revealed differences in the morphology of the geopolymer matrix depending on the composition of the mixtures. The reference sample (100FA, Figure 22) showed a heterogeneous, amorphous matrix with numerous irregularly shaped pores. At higher magnifications, denser and more loosely packed areas were observed, with microcracks running through the binder phase. These features are similar to those described in the literature for alkali-activated aluminosilicate gels, where partial geopolymerization can lead to local porosity [1,76].

**Figure 23:**
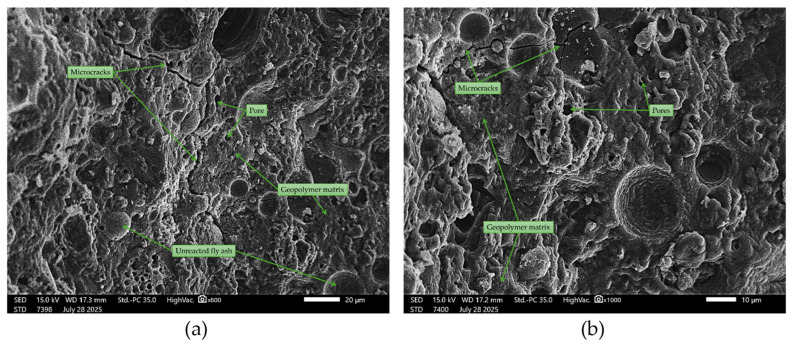
SEM images of geopolymer with 50% quartz sand (50FA + 50S): (**a**) ×600 magnification; (**b**) ×1000 magnification.

Samples containing quartz sand (Figure 23) exhibited a more complex microstructure, with clearly visible sand grains partially embedded in the geopolymer matrix. The interface between the sand and the binder was not always continuous, and in some areas, small interfacial gaps formed, likely due to uneven shrinkage during the curing process. The binder in these samples retained its amorphous character but showed local densification around the sand particles. Many sources indicate that such interfacial features affect mechanical strength and durability [77,78].

**Figure 24:**
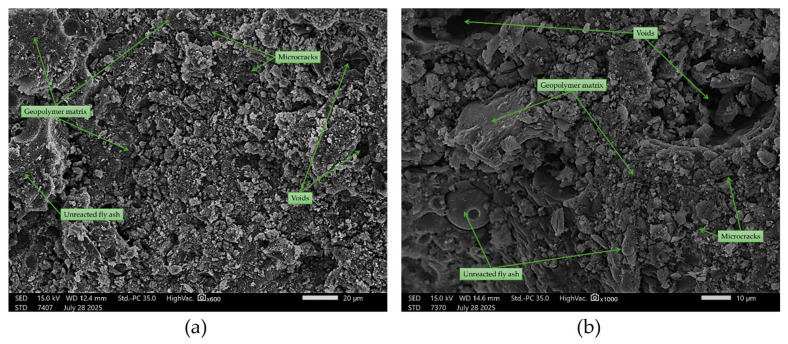
SEM images of geopolymer with 50% reclaimed asphalt (50FA + 50A): (**a**) ×600 magnification; (**b**) ×1000 magnification.

The addition of asphalt (Figure 24) introduced an additional phase with a significantly smoother texture compared to the geopolymer matrix. This phase often fills pores and microvoids, resulting in a reduction in overall porosity. In some areas, this phase filled pores and microvoids, thereby reducing local porosity. Cases of tight adhesion of asphalt to the geopolymer were observed, resulting in continuous films within the binder in certain areas. This type of interfacial contact may reduce water absorption.

**Figure 25:**
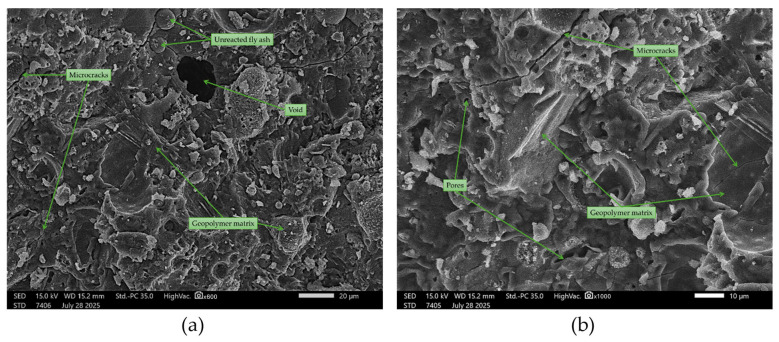
SEM images of ternary composite materials (50FA + 25S + 25A): (**a**) ×600 magnification; (**b**) ×1000 magnification.

**Figure 26:**
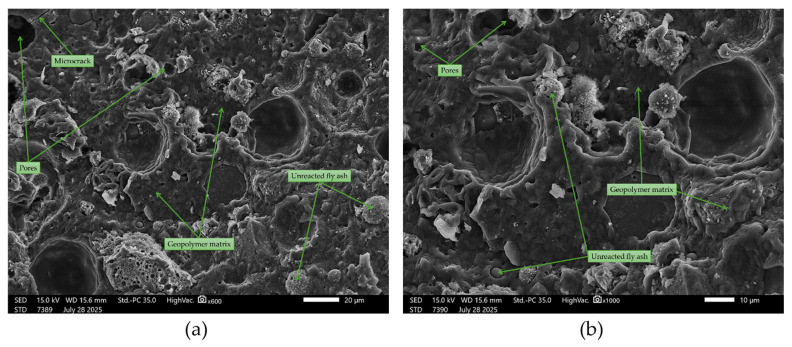
SEM images of ternary composite materials (33FA + 33S + 33A): (**a**) ×600 magnification; (**b**) ×1000 magnification.

In the ternary composite materials (sand + asphalt + fly ash), the microstructure in the observed areas appeared more compact and homogeneous (Figure 25 and Figure 26). The asphalt phase filled some of the gaps at the sand–binder interface, and the number of visible microcracks was lower than in the other variants. Smoother interphase transitions and fewer voids in the structure were observed, which may indicate a beneficial effect of the sand and asphalt combination. Similar effects of matrix densification and improved interfacial contact were noted in other geopolymer systems with complex structures [79].

It is essential to emphasize that the fly ash-based geopolymer gel remains the primary binding phase in all mixtures. At the same time, sand and asphalt primarily serve as fillers, modifying their structure. The quartz sand particles, being chemically inert, do not participate in the geopolymerization reaction; however, they enhance the packing density and reduce porosity, a phenomenon also reported in studies by Zawrah et al. [61] and Gao et al. [59]. In contrast, the milled asphalt introduces a significant fraction of non-reactive organic and carbonate phases, which may partially block the continuity of the geopolymer gel and decrease the degree of polymerization. Therefore, the differences in strength and durability cannot be interpreted as equivalent matrices of different compositions, but rather as geopolymer matrices of varying integrity, modified by inert or partially disruptive fillers. The matrix–filler interactions thus play a decisive role in shaping the final composite performance.

## 4. Conclusions

This study aimed to evaluate the influence of quartz sand and milled asphalt on the physical, mechanical, and structural properties of fly ash-based geopolymer composites, with a particular focus on their potential application in road construction. Based on the experimental results and analyses presented, the following conclusions can be drawn:

- The addition of quartz sand (S) contributed to improved key material parameters. This filler increased density and compressive strength while reducing water absorption and abrasion susceptibility, which can be attributed to enhanced grain packing and the favorable role of the silica phase in the cross-linking process of the geopolymer matrix.
- Milled asphalt (A), acting primarily as a passive filler, decreased mechanical strength at higher dosages. At the same time, when combined with sand, it limited water sorption due to its hydrophobic character, which may be advantageous in selected applications.
- Fly ash (FA) remained the main reactive component in the geopolymerization process. Its proportion determined both the reaction progress and the durability of the composites. Reducing FA content restricted the amount of aluminosilicate precursors, resulting in lower strength and modifications to the internal structure.
- Ternary composite materials, especially the 33FA + 33S + 33A variant, offered a balanced compromise between strength, dimensional stability, and moisture resistance, confirming the benefits of properly selected component ratios.
- The study also demonstrates that the geopolymer matrix based on fly ash is the dominant factor governing the overall performance of the composites. Additives such as sand or milled asphalt cannot be considered equivalent replacements for the geopolymer gel; instead, they act as modifying phases that influence density, porosity, and water uptake. Quartz sand enhanced densification and strength, while asphalt, despite reducing compressive strength at high contents, contributed to lower water sorption. These results highlight that the observed effects arise from the interaction between the geopolymer gel and the fillers, and not from equivalent matrices with altered compositions, which is crucial for assessing their practical applicability.

Based on these findings, further research is recommended in three directions:
  - limiting the milled asphalt content to a maximum of 25% to minimize loss of strength,
  - improving asphalt fragmentation to obtain a more uniform structure and reduce internal defects,
  - and testing higher molarity activators or additional reactive precursors such as metakaolin to enhance polycondensation and improve the mechanical performance of the composites.

## Figures and Tables

**Table 1:** Marking of samples depending on the type and amount of sand and asphalt additives introduced from the demolition of road surfaces.

Sample Designation	Weight Fraction [%wt.]			The Liquid-to-Solid (L/S) Ratio
	Fly Ash	Sand	Asphalt	
100FA	100	–	–	0.4
75FA + 25S	75	25	–	0.4
75FA + 25A	75	–	25	0.425
50FA + 50S	50	50	–	0.4
50FA + 50A	50	–	50	0.45
50FA + 25S + 25A	50	25	25	0.425
33FA + 33S + 33A	33	33	33	0.425

**Table 2:** Material abrasion classes [43].

Abrasion Resistance Class	Average Height Loss [mm]
Very high abrasion	>10.0
High abrasion	7.5–10.0
Low abrasion	2.5–5.0
Very low abrasion	<2.5

**Table 3:** Particle size distribution data for fly ash.

Name	D_10_ [µm]	D_50_ [µm]	D_90_ [µm]	Mean Size [µm]	Span Index
Mean value	2.709	16.532	50.786	23.59	2.908
Standard deviation	0.047	0.073	0.946	0.247	0.061
Relative standard deviation [%]	1.74	0.44	1.86	1.05	2.09

**Table 4:** Particle size distribution data for sand.

Name	D_10_ [µm]	D_50_ [µm]	D_90_ [µm]	Mean Size [µm]	Span Index
Mean value	182.57	341.715	474.501	374.146	0.854
Standard deviation	29.727	6.227	3.108	5.918	0.087
Relative standard deviation [%]	16.28	1.82	0.66	1.58	10.21

**Table 5:** Particle size distribution data for asphalt.

Name	D_10_ [µm]	D_50_ [µm]	D_90_ [µm]	Mean Size [µm]	Span Index
Mean value	172.948	250.576	437.076	311.536	1.177
Standard deviation	74.589	93.463	73.374	88.079	0.481
Relative standard deviation [%]	43.13	37.3	16.79	28.27	40.87

**Table 6:** One-way ANOVA—flexural strength.

Source of Variation	Sum of Squares SS	Degrees of Freedom df	Mean Square MS	Fisher’s Test Value F	Significance Level *p*-Value	Critical F Value Fcrit
Between groups	94.76	6	15.79	35.90	6.79·× 10^−12^	2.45
Within groups	12.32	28	0.444			
Total	107.08	34				

**Table 7:** One-way ANOVA—compressive strength.

Source of Variation	Sum of Squares SS	Degrees of Freedom df	Mean Square MS	Fisher’s Test Value F	Significance Level *p*-Value	Critical F Value Fcrit
Between groups	5017.522	6	836.25	28.83	9.44·× 10^−11^	2.45
Within groups	812.254	28	29.01			
Total	5829.77	34				

**Table 8:** Comparison of the elemental composition of geopolymer samples with different proportions of fly ash, sand, and milled asphalt—results from XRF analysis.

Element	Concentration [%]						
	100FA	75FA + 25S	75FA + 25A	50FA + 50S	50FA + 50A	50FA + 25S + 25A	33FA + 33S + 33A
Mg	1.94	4.39	1.54	0.9	1.97	0.00	1.27
Al	13.81	12.11	11.27	10.88	13.34	9.41	10.14
Si	48.22	44.54	47.19	49.37	48.01	42.74	46.29
P	0.00	0.00	0.00	0.23	0.00	0.05	0.22
S	0.69	0.39	0.9	3.89	0.26	11.09	3.75
Cl	0.12	0.11	0.11	0.07	0.13	0.05	0.08
K	6.8	7.36	7.25	7.25	6.74	7.2	7.61
Ca	7.26	7.58	8.14	7.14	8.68	7.97	9.58
Ti	2.02	2.21	2.16	1.97	2.06	2.03	2.00
V	0.11	0.12	0.12	0.17	0.1	0.17	0.19
Cr	0.04	0.05	0.05	0.04	0.04	0.05	0.05
Mn	0.3	0.34	0.33	0.34	0.29	0.33	0.38
Fe	17.53	19.56	19.63	16.84	17.29	17.82	17.35
Ni	0.05	0.06	0.06	0.05	0.05	0.06	0.06
Cu	0.07	0.09	0.08	0.07	0.07	0.08	0.08
Zn	0.11	0.13	0.16	0.11	0.16	0.14	0.23
Ga	0.02	0.02	0.02	0.02	0.02	0.02	0.02
As	0.02	0.03	0.03	0.03	0.02	0.04	0.04
Br	0.01	0.01	0.01	0.00	0.01	0.00	0.00
Rb	0.07	0.08	0.08	0.05	0.06	0.06	0.05
Sr	0.22	0.23	0.24	0.16	0.2	0.2	0.17
Y	0.02	0.01	0.01	0.00	0.01	0.01	0.00
Zr	0.13	0.14	0.14	0.1	0.11	0.12	0.1
Rh	0.00	0.00	0.00	0.00	0.02	0.00	0.00
In	0.00	0.04	0.05	0.00	0.03	0.00	0.00
Ba	0.42	0.38	0.37	0.32	0.31	0.35	0.36
Ta	0.00	0.00	0.02	0.00	0.02	0.00	0.00
Pb	0.01	0.01	0.02	0.00	0.02	0.00	0.00

**Table 9:** Comparison of the oxide composition of geopolymer samples with different proportions of fly ash, sand, and milled asphalt—results from XRF analysis.

Oxide	Concentration [%]						
	100FA	75FA + 25S	75FA + 25A	50FA + 50S	50FA + 50A	50FA + 25S + 25A	33FA + 33S + 33A
MgO	0.987	3.675	1.728	0.252	0.936	0.00	0.00
Al_2_O_3_	16.721	14.298	13.512	12.761	15.992	11.126	12.417
SiO_2_	60.331	57.2	60.068	61.259	60.423	53.694	59.328
P_2_O_5_	0.00	0.00	0.00	0.271	0.00	0.092	0.281
SO_3_	0.85	0.51	1.12	4.74	0.34	13.59	4.77
Cl	0.052	0.054	0.05	0.038	0.068	0.03	0.039
K_2_O	3.9	4.46	4.25	4.13	3.97	4.03	4.43
CaO	4.66	5.13	5.3	4.54	5.67	4.99	6.19
TiO_2_	1.5	1.72	1.63	1.45	1.55	1.48	1.49
V_2_O_5_	0.08	0.09	0.09	0.13	0.08	0.13	0.14
Cr_2_O_3_	0.02	0.03	0.03	0.02	0.03	0.02	0.02
MnO	0.15	0.19	0.18	0.18	0.16	0.17	0.2
Fe_2_O_3_	10.2	12.06	11.5	9.83	10.32	10.18	10.19
NiO	0.02	0.03	0.02	0.02	0.02	0.02	0.02
CuO	0.03	0.04	0.03	0.03	0.03	0.03	0.03
ZnO	0.05	0.06	0.07	0.05	0.08	0.06	0.1
Ga_2_O_3_	0.01	0.01	0.01	0.01	0.01	0.01	0.00
As_2_O_3_	0.01	0.01	0.01	0.02	0.01	0.02	0.02
Rb_2_O	0.03	0.04	0.04	0.02	0.03	0.03	0.02
SrO	0.1	0.11	0.11	0.07	0.09	0.09	0.08
Y_2_O_3_	0.01	0.01	0.01	0.00	0.01	0.00	0.00
ZrO_2_	0.06	0.07	0.07	0.05	0.05	0.06	0.05
BaO	0.17	0.16	0.15	0.13	0.13	0.13	0.14

## Data Availability

The original contributions presented in this study are included in the article. Further inquiries can be directed to the corresponding author.
